# Genotype-driven cerebrovascular risk across age and sex in hereditary hemorrhagic telangiectasia

**DOI:** 10.1007/s10072-026-09018-z

**Published:** 2026-04-16

**Authors:** Matteo Palermo, Federico Cocilovo, Gianluca Trevisi, Emanuela Lucci Cordisco, Luigi Di Martino, Elena Sonnini, Alessio Albanese, Francesco Doglietto, Alessandro Olivi, Roberto Pola, Eleonora Gaetani, Carmelo Lucio Sturiale

**Affiliations:** 1 Department of Neurosurgery, Fondazione Policlinico Universitario A. Gemelli IRCCS, Università Cattolica del Sacro Cuore, Rome, Italy; 2 Department of Neurosciences, Imaging and Clinical Sciences, G. D’Annunzio University, Chieti-Pescara, Italy; 3 Dipartimento di Scienze della Vita e Sanità Pubblica, UOC Genetica Medica, Fondazione Policlinico Universitario A. Gemelli IRCCS, Università Cattolica del Sacro Cuore, Rome, Italy; 4 Department of Translational Medicine and Surgery, Fondazione Policlinico Universitario A. Gemelli IRCCS Università Cattolica del Sacro Cuore, Rome, 00168 Italy; 5 Unit of Internal Medicine, Cristo Re Hospital, Rome, 00167 Italy; 6 Department of Aging, Orthopedic, and Rheumatologic Sciences, Fondazione Policlinico Universitario A. Gemelli IRCCS, Università Cattolica del Sacro Cuore, Rome, 00168 Italy; 7 Institute of Neurosurgery, Fondazione Policlinico Universitario “A. Gemelli” IRCCS, Università Cattolica del Sacro Cuore, L.go A. Gemelli 8, Rome, 00168 Italy

## Abstract

**Background:**

Hereditary hemorrhagic telangiectasia (HHT) is a rare autosomal dominant vascular disorder characterized by multisystem arteriovenous malformations (AVMs). Still, the influence of demographic factors and specific pathogenic variants on systemic and cerebrovascular involvement remains incompletely defined.

**Methods:**

We retrospectively included 142 patients with genetically confirmed HHT diagnosis. Systemic and neurovascular data were collected for each patient and stratified by age and sex. Then, we conducted univariate analyses for genotype-phenotype correlations after adjusting for age and sex. Afterwards, the significant associations were tested in multivariable logistic regression models to verify confounding effects.

**Results:**

Hepatic AVMs and gastrointestinal bleeding increased significantly with age, whereas neurological manifestations showed no age dependency. In multivariable analysis for hepatic AVMs, increasing age was independently associated with hepatic involvement (*p* = 0.037), while ENG (*p* = 0.035) was associated with a lower likelihood of hepatic AVMs compared to ACVRL1. For brain AVMs, age, ENG gene, and variant truncation status were independent predictors, and the ACVRL1 c.277 C > T (p.Arg93*) mutation showed an independent association (*p* = 0.035).

**Conclusions:**

In HHT, age and gene-level effects are robust predictors of systemic AVM involvement, whereas mutation-specific associations remain difficult to evaluate. Larger multicenter studies are needed to validate variant-level risk and support personalized surveillance strategies.

## Introduction

Hereditary hemorrhagic telangiectasia (HHT) is an inherited vascular disorder characterized by multisystemic arteriovenous malformations involving the pulmonary, hepatic, and central nervous systems [1, 2]. Cerebrovascular manifestations represent a major determinant of clinical outcome, encompassing brain and spinal arteriovenous malformations, intracranial hemorrhage, epilepsy, and ischemic events [1–3]. Despite their clinical relevance, the factors driving the marked heterogeneity of cerebrovascular involvement in HHT remain incompletely understood.

This heterogeneity likely reflects the interaction between underlying genetic determinants and host-related biological modifiers that influence vascular development and remodeling over time [4, 5]. Pathogenic variants in ENG, ACVRL1, and SMAD4 disrupt key endothelial signaling pathways, yet patients carrying identical mutations may exhibit strikingly different cerebrovascular phenotypes, suggesting that genetic background alone is insufficient to explain disease expression [1]. Age-related vascular maturation and cumulative remodeling, as well as sex-related differences in endothelial biology and angiogenic signaling, are plausible contributors to this variability and may shape both lesion penetrance and clinical presentation [6–9].

Previous studies have often addressed cerebrovascular manifestations in HHT by focusing on either genetic background or clinical features, without fully contextualizing cerebrovascular phenotypes within the broader biological framework in which demographic factors act as intrinsic modifiers of disease expression [1, 2, 10]. As a result, the relative contribution of genotype and host characteristics to cerebrovascular heterogeneity remains difficult to disentangle, and clinically meaningful patterns may be obscured. Importantly, prior investigations have been constrained by several inherent methodological limitations, including small single-center cohorts, frequent familial clustering that complicates genotype–phenotype interpretation, and low event rates for specific cerebrovascular outcomes. These factors reduce statistical power, limit generalizability, and increase the risk of both type II error and overinterpretation of isolated associations.

In this study, we investigated cerebrovascular manifestations in HHT within an integrated biological framework that considers genetic background alongside age- and sex-related modifiers of vascular phenotype. By examining cerebrovascular features across different demographic strata and genetic subgroups, we aimed to provide a coherent characterization of the factors shaping cerebrovascular disease expression in HHT and to clarify how genetic susceptibility and host biology jointly influence neurovascular involvement.

## Methods

### Study design and patient cohort

One hundred-forty-two patients with confirmed HHT diagnosis were retrospectively analyzed and included in the study. The Curaçao score, including epistaxis, mucocutaneous telangiectasias, visceral involvement, and family history, was utilized to confirm the diagnosis (Table 1). Each patient was identified through a dedicated institutional registry. Demographics (age and sex), family history, and detailed clinical data on HHT-related manifestations were collected for each patient. Radiological data from CT, MRI and ultrasound were examined to evaluate the extent of organ-involvement. We evaluated the presence of pulmonary, hepatic, and cerebral arteriovenous malformations (AVMs). Brain scans were used to obtain data on arteriovenous fistulas (AVFs), intracranial aneurysms (IAs), cerebral cavernous malformations (CCMs), and developmental venous anomalies (DVAs). In addition, cerebral abscesses and occurrences of ischemic or hemorrhagic stroke were recorded. Patients underwent genetic testing to identify pathogenic variants in *ENG*, *ACVRL1*, or *SMAD4*. Then, transcripts were classified as truncating or non-truncating.

Ethical approval was required for this study (IRB: 49901/18: ID 2329). All analyses were performed in accordance with institutional ethical standards.

**Table 1:** Curaçao diagnostic criteria for HHT

Domain	Clinical Feature	Definition	Score
Clinical bleeding	Recurrent epistaxis	Spontaneous and recurrent nosebleeds	0–1
Cutaneous/mucosal lesions	Telangiectasias	Multiple telangiectasias at characteristic sites (lips, oral cavity, fingers, nose)	0–1
Visceral involvement	AVMs or GI telangiectasias	Pulmonary, cerebral, spinal, hepatic AVMs or gastrointestinal lesions	0–1
Family history	First-degree relative	Confirmed HHT in a first-degree relative	0–1
	Total score	Sum of criteria (0–4)	**0–4**
Interpretation	Definite HHT	≥ 3 criteria present	
	Possible HHT	2 criteria present	
	Unlikely HHT	≤ 1 criterion present	

### Statistical analysis

Initially, patients were stratified into three age groups: young adults (20–40 years), adults (40–60 years), and older adults (> 60 years). All patients were older than 20. Statistical analyses were conducted using JASP software (version 0.95.3; September 30th, 2025). A p-value < 0.05 was considered statistically significant.

Comparisons across age groups were performed using the chi-square test for categorical variables and the Kruskal–Wallis test for ordinal variables. Patient distribution was non-parametric. Gender analyses were done using Fisher’s exact test.

The second step involved running univariate analyses to assess associations between individual pathogenic variants and cerebrovascular malformations. Then, to account for potential familiar clustering, we fitted a generalized linear mixed model (GLMM) with “Family” as a random effect. The model proved non-convergent because most families were represented by a single individual. Thus, multivariable logistic regression models were employed.

Multivariable logistic regression models were constructed for cases showing statistical significance in univariate analyses. Covariates included sex, age, affected gene, transcript type, and the specific mutation(s) identified in the univariate analysis.

To limit model overfitting, Model 0 was restricted to age and sex. Separate multivariable models were generated for each outcome to evaluate potential confounding effects.

## Results

A total of 413 patients with a confirmed diagnosis of HHT were retrospectively identified from the HHT registry. Because brain MRI is not yet a mandatory screening examination for all HHT patients, only subjects with an available brain MRI on December 2nd, 2025, were eligible for the present analysis, resulting in a final cohort of 142 patients. All patients underwent genetic testing to molecularly confirm the diagnosis. Analyses stratified by age and sex are reported below. Genotype–phenotype correlations are presented in the corresponding section. All variables of interest were fully collected for every included patient without missing information.

## Age-related cerebrovascular phenotypes

Thirty-nine young adults (27.21 ± 8.13 years), 69 adults (52.58 ± 7.25 years), and 34 older patients (71.97 ± 4.62 years) were included (*p* < 0.001). Sex distribution was evenly distributed across age groups (*p* = 0.221). The pathogenic gene variant distribution did not differ significantly with age (ACVRL1, *p* = 0.330; ENG, *p* = 0.638; SMAD4, *p* = 0.267). Curaçao scores did not differ by age (*p* = 0.056). Patients in all groups presented a relevant family history of HHT: 37/39 young, 62/69 adults, and 34/34 old patients had an affected relative (*p* = 0.130).

The prevalence of cutaneous telangiectasia rose with age, observed at 27 young (69%), 59 adult (86%), and 32 old patients (94%; *p* = 0.014). The rate of pulmonary AVMs and epistaxis was similar across age groups (*p* = 0.799, *p* = 0.857). Gastrointestinal bleeding was more frequent in older individuals (*p* = 0.032). Hepatic AVMs were also significantly more common with increasing age: 7 young, 32 adults, 15 old (*p* = 0.010).

The occurrence of neurological complications did not differ significantly among age categories. There were no statistically significant differences in brain vascular lesions between age groups (Table 2).

**Table 2:** Analysis of HHT manifestations stratified by age

Age (SD)	Young Adult (*n* = 39)	Adult (*n* = 69)	Old (*n* = 34)	*p*-value
	27.21 (8.125)	52.58 (7.253)	71.97 (4.615)	< 0.001
Sex				0.221
F	18	14	40	
M	21	20	29	
Gene				
ACVRL1	19	42	22	0.330
ENG	17	24	12	0.638
SMAD4	3	3	0	0.267
Curacao				0.056
I	2	0	0	
II	5	6	0	
III	17	25	14	
IV	15	38	20	
Familiarity	37	62	34	0.130
Organ-specific findings				
Cutaneous telangiectasia	27	59	32	**0.014**
Epistaxis	36	67	33	0.799
Pulmonary AVM	15	24	11	0.857
GI bleeding	2	16	9	**0.032**
Hepatic AVM	7	32	15	**0.010**
Neurological Dysfunction				
Epilepsy	4	5	1	0.474
Focal Deficit	4	4	5	0.324
Headache	5	8	2	0.584
Hemorrhage	1	0	0	0.595
Brain finings				
bAVM	9	12	2	0.129
Aneurysm	0	2	2	0.317
Cavernoma	1	3	0	0.453
dAVF	0	0	1	0.202
DVA	2	3	1	0.896
Telangiectasia	1	2	0	0.613
Stroke	3	8	4	0.791
Abscess	2	2	1	0.815

## Sex-related cerebrovascular phenotypes

There were 72 female and 70 male patients with no age difference (*p* = 0.546) (Table 3). Genotype frequencies were comparable between sexes: ACVRL1 mutations (*p* = 0.736), ENG (*p* = 0.762), and SMAD4 (*p* = 0.102). The distribution of Curaçao criteria fulfillment was similar (*p* = 0.887). No significant differences in organ involvement were observed.

**Table 3:** Analysis of HHT manifestations stratified by sex

Age	Female (*n* = 72)	Male (*n* = 70)	*p*-value
	49.92 (16.61)	51.21 (18.75)	0.546
Gene			
ACVRL1	41	42	0.736
ENG	26	27	0.762
SMAD4	5	1	0.102
Curacao			0.887
I	38	35	
II	65	68	
III	61	57	
IV	70	66	
Familiarity	65	68	0.166
Organ-specific findings			
Cutaneous telangiectasia	61	57	0.658
Epistaxis	70	66	0.209
Pulmonary AVM	25	25	1.00
GI bleeding	13	14	0.832
Hepatic AVM	30	24	0.392
Neurological Dysfunction			
Epilepsy	2	5	0.272
Focal Deficit	2	11	**0.009**
Headache	12	11	0.587
Hemorrhage	3	1	0.272
Brain finings			
bAVM	12	11	1.00
Aneurysm	3	1	0.620
Cavernoma	3	0	0.120
dAVF	1	0	1.00
DVA	3	3	0.972
Telangiectasia	3	0	0.245
Stroke	6	9	0.424
Abscess	1	4	0.206

Most neurological manifestations were equally common in women and men, with one exception. Notably, focal neurological deficits were more frequent in males (11 of 70, 15.7%) than females (2 of 72, 2.8%; *p* = 0.009).

There were no significant sex differences in brain vascular lesions.

### Genotype–phenotype associations adjusted by age and sex

Several specific mutations showed significant associations or trends with clinical phenotypes in univariate analysis (Table 4):

**Table 4:** Bivariate genotype-phenotype analysis with significant and borderline significant results

Phenotype	Mutation	Odds Ratio	95% CI	*p*-value
Cavernoma	c.1245_1248del p.Asp415GlufsTer20	117.9	4.05–3433.47	**0.028**
Brain AVM	c.277 C > T (p.Arg93*)	17.7	1.75–178.72	**0.014**
Brain aneurysm	c.400_409delGCCCTGGGTG	118.7	4.08–3458.33	**0.028**
Brain aneurysm	c.1232G > A (p.Arg411Gln)	22.67	1.59–323.76	0.083
Brain aneurysm	c.1435 C > T (p.Arg479*)	18.71	2.29–153.17	**0.02**
Pulmonary AVM	c.1120 C > T (p.Arg374Trp)	0.17	0.02–1.35	0.097
GI bleeding	c.1232G > A (p.Arg411Gln)	9.12	0.80–104.55	0.093
DVA	c.1080 ins. dupl (19 bp)	74.45	2.71–2043.38	**0.042**
DVA	c.971 C > G (p.Ser324Ter)	74.45	2.71–2043.38	**0.042**
Brain Capillary telangiectasia	c.986G > A	167.4	5.39–5201.31	**0.021**
Brain Capillary telangiectasia	c.250delG	34.25	2.13–550.35	**0.062**
Hepatic AVMs	c.197 A > C (p.His66Pro)	15.77	0.83–298.98	**0.019**
Hepatic AVMs	c.1232G > A (p.Arg411Gln)	12.03	0.61–237.55	0.053
Hepatic AVMs	c.309_311del (p.Ser104del)	0.087	0.005–1.54	**0.024**

The ENG mutation c.1245_1248del (p.Asp415GlufsTer20) was associated with the presence of a CCMs, with an odds ratio (OR) of 117.9 (*p* = 0.028). The ACVRL1 nonsense mutation c.277 C > T (p.Arg93*) was associated with bAVM (OR 17.7, *p* = 0.014). Two mutations in ENG were significantly linked to IAs: the deletion c.400_409delGCCCTGGGTG showed a strong association (*p* = 0.028), and the nonsense mutation c.1435 C > T (p.Arg479*) (OR 18.71, *p* = 0.020). Two mutations were significantly associated with the presence of a DVA: an ENG frameshift insertion/duplication and the ENG nonsense mutation c.971 C > G (p.Ser324Ter). The ENG missense mutation c.986G > A was associated with brain capillary telangiectasia (OR 167.4, *p* = 0.021).

In ACVRL1, the missense mutation c.197 A > C (p.His66Pro) was associated with increased odds of hepatic AVMs (OR 15.77, *p* = 0.019). Two ENG variants also showed high odds for hepatic AVMs: c.1232G > A (p.Arg411Gln) with OR 12.03 (*p* = 0.053, borderline) and c.309_311del (p.Ser104del), which was associated with significantly lower odds (OR 0.087, 95% CI 0.005–1.54, *p* = 0.024).

Each of the significant genotype-phenotype associations were then tested in the multivariate models reported in the following paragraph.

## Multivariate models

The multivariate model (Table 5) showed that age and sex did not reach significance. Using the third gene as dummy variable, ENG was significantly associated with increased odds of bAVM (OR = 20.406, *p* < 0.001). Additionally, patients with truncating mutations had lower odds of bAVM than those with non-truncating variants (OR -1.339, *p* = 0.027). Additionally, the specific *ENG c.277 C > T (p.Arg93) variant* included in the model showed a large effect size (OR 15.166, *p* = 0.035).

**Table 5:** Resluts of the multivariate logistic regression for bAVM

Predictor	β (SE)	Odds Ratio (OR)	95% CI for OR	*p*-value
Age	-0.030 (0.016)	0.970	0.940–1.001	0.061
Sex (Male vs. Female)	0.361 (0.578)	1.435	0.462–4.457	0.532
Gene (ENG vs. Other)	3.016 (0.739)	20.406	4.793–86.876	**< 0.001**
Gene (SMAD4 vs. Other)	2.069 (1.325)	7.913	0.589–106.236	0.119
Variant (Truncated Vs Non-Truncated)	-1.339 (0.604)	0.262	0.080–0.856	**0.027**
c. 277 C > T (p.R93X)	2.719 (1.292)	15.166	1.206–190.683	**0.035**

In multivariable logistic regression analyses assessing predictors of hepatic AVMs, the model became unstable when the individual mutation that was significant at univariate analysis was included. This instability was due to sparse data and quasi-complete separation. Therefore, the individual mutation was excluded, and the multivariable model was re-run.

In the final model, increasing age (OR = 1.026) was independently associated with hepatic AVMs (*p* = 0.040). ENG mutations were associated with a significantly lower likelihood of hepatic AVMs compared to ACVRL1 (OR = 0.353; *p* = 0.022). Curaçao Score was a significant prediction (*p* < 0.001). Differently, both transcripts and protein’s domains affected were not significant in predicting occurrence of hepatic AVMs.

The multivariable model for brain aneurysms, DVAs, and brain capillary telangiectasia were largely unstable when entering the mutation found during the univariate analysis. This was due to sparse data bias and quasi-complete separation. Likewise, as done for hepatic AVMs, attempts at running the multivariate model without the variant were made but remained unsuccessful. Therefore, these models were considered unreliable and were not interpreted.

## Discussion

### Genotype–phenotype correlations

Understanding how specific genetic variants translate into distinct cerebrovascular phenotypes remains a central challenge in HHT, with direct implications for risk stratification, surveillance strategies, and personalized management.

In this context, our study identified several genotype–phenotype associations. Although multiple associations were observed in univariate analyses, many of these did not remain statistically significant after adjustment for potential confounding factors in multivariable models aligning with what reported by Kasthuri et al. [11]. Notably, the association between the ENG nonsense variant c.277 C > T (p.Arg93)* and the presence of brain AVMs persisted after multivariable adjustment (*p* = 0.035). In the same model, transcript type was also independently associated with brain AVMs (*p* = 0.027).

The ENG c.277 C > T (p.Arg93*) mutation is a known recurrent “hotspot” in HHT1 families [12, 13], and our data suggest it may confer especially high risk for brain AVMs.

Despite the inability to adjust familial clustering that led us to abandon the GLMM and opt for a standard multivariable logistic regression model, none of the patients carrying this mutation, who were reported to have bAVMs, were related. These findings argue against familial aggregation as the primary driver of this association. Still, the wide confidence interval, largely unavoidable in rare diseases, warns for cautious interpretation and warrants further investigation in larger series.

To our knowledge, this specific mutation-level association has not been previously reported in the literature. However, these findings should be interpreted in light of the limitations of our analysis.

Likewise, the ACVRL1 c.1435 C > T variant may be associated with a distinct cerebrovascular risk profile. A recent study reported that approximately 75% of patients carrying this mutation had cerebrovascular abnormalities, including AVMs and IAs, a rate well above that expected in the general HHT population [5]. This observation is in line with our finding of IAs in patients with this variant, even though the association did not remain statistically significant after multivariable adjustment. Additional studies are needed to clarify this relationship and to determine whether the lack of significance in our multivariable analysis may be related to residual confounding, particularly due to familial clustering that could not be fully addressed in the present study.

Broadly, our data confirm the well-established patterns reported by Lesca et al. [14]. ENG (HHT1) mutation carriers are prone to pulmonary and cerebral AVMs (*p* < 0.001), whereas ACVRL1 (HHT2) carriers more often have hepatic AVMs and later-onset GI bleeding [14] . Similarly, Bayrak-Toydemir et al. reported that cerebral AVMs were more frequent in HHT1, whereas hepatic vascular malformations were largely present in HHT2 patients [12]. Future studies should explore whether proactive, genotype-informed screening across multiple vascular territories may offer clinical advantages over the current reactive approach [11].

### Molecular and pathway implications of genotype–phenotype associations

**Fig. 1:**
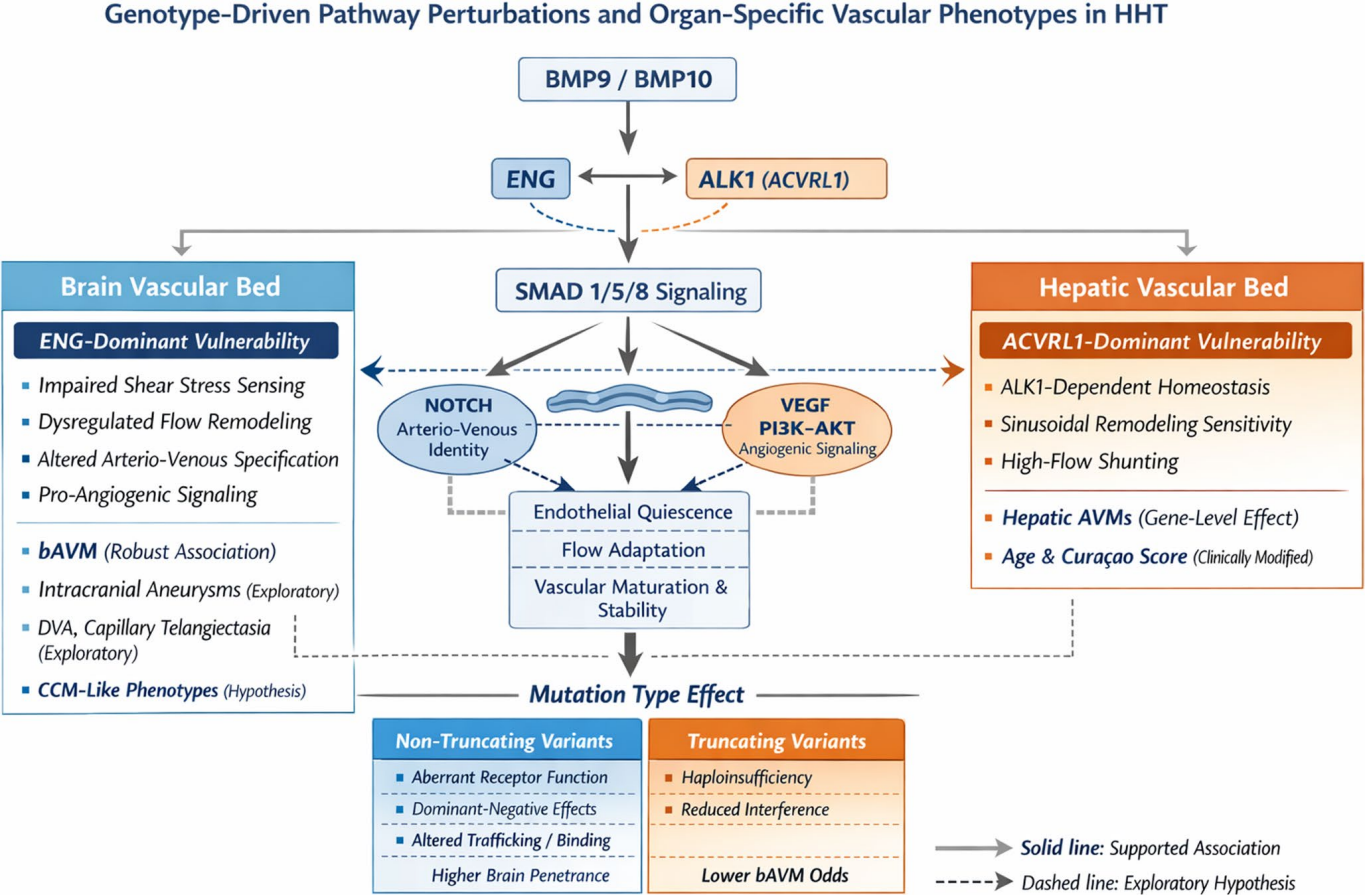
Genotype-driven pathway perturbations and organ-specific vascular phenotypes in HHT

The observed genotype–phenotype associations point toward a biologically coherent involvement of the endothelial BMP9/10–ALK1–endoglin signaling axis in shaping organ-specific vascular vulnerability [15]. In the multivariable model adjusted for age and sex, ENG mutations was independently associated with a markedly increased likelihood of bAVMs (Table 5**)**, whereas ENG variants were associated with a significantly lower probability of hepatic AVMs compared with ACVRL1 (Table 6), which may hypothetically reflect a bed-specific heterogeneity of signaling dependency across vascular territories (Fig. 1). Endoglin plays a central role in endothelial mechano-transduction and modulation of shear-stress–responsive pathways, as well as in the fine-tuning of SMAD1/5/8 activation downstream of BMP9/10 [15, 16]. Impaired ENG-mediated signaling may therefore preferentially disrupt flow-dependent vascular remodeling and arterio-venous specification in the cerebral circulation, promoting maladaptive angiogenesis and shunt formation [16]. In contrast, hepatic vascular remodeling has been shown experimentally to be related with ALK1-mediated signaling, possibly in line with the classical association between ACVRL1 variants and hepatic involvement [17]. In line with recent evidence, Kasthuri et al. [11] reported that despite intragenomic-individual differences, these genotype–phenotype analysis of prevalence has limited utility for risk stratification or clinical management [11]. Environmental and inflammatory factors further modulate disease severity and vascular expression [11]. Consequently, gene-based surveillance protocols have not been recommended despite observed phenotypic trends.

**Table 6:** Results of the multivariate logistic regression for hepatic AVM

Predictor	β (SE)	Odds Ratio (OR)	95% CI for OR	*p*-value
Age	0.026 (0.013)	1.026	1.001–1.051	**0.040**
Sex (Male vs. Female)	-0.279 (0.398)	0.757	0.347–1.650	0.483
Gene (ENG vs. Others)	-1.040 (0.455)	0.353	0.145–0.862	**0.022**
Gene (SMAD4 vs. Others)	1.587 (0.994)	4.890	0.697–34,296	0.110
Curaçao Score	1.509 (0.392)	4.524	2.099–9.750	**< 0.001**
Variant (Truncated vs. Non-Truncated)	0.232 (0.419)	1.261	0.555–2.865	0.579

Interestingly, truncating variants were associated with lower odds of bAVMs compared with non-truncating mutations. While truncating and frameshift variants have been shown in cancer to induce nonsense-mediated mRNA decay and reduced transcript abundance, whether this mechanism applies in non-neoplastic vascular disease remains to be established [18, 19]. Therefore, beyond effects on endoglin protein translation or processing, these mutations may also compromise mRNA stability [18, 20], thus possibly causing effects on the receptor complex [21]. Such qualitative disruptions may impose a stronger pathogenic burden within the cerebral microvascular environment than simple dosage reduction alone, potentially explaining the higher bAVM risk observed in carriers of non-truncating variants. This observation, although requiring cautious interpretation given the limitations of the sample size, is consistent with emerging concepts of mutation-specific functional heterogeneity within the TGF-β superfamily signaling network.

Variant-level associations with IAs, DVAs, brain capillary telangiectasia, and CCMs were observed in univariate analyses, but multivariable models were unstable due to small sample size and sparse data. Nonetheless, these exploratory signals raise the hypothesis that selected ENG variants may converge on pathways regulating microvascular stability or extracellular remodeling, like KLF2/KLF4 [22] or NOTCH–VEGF^23^, thereby predisposing to a broader spectrum of cerebrovascular phenotypes beyond classical AVMs [1].

Future functional studies integrating endothelial signaling assays, shear-stress–responsive transcriptional profiling, and variant-specific mechanistic modeling will be essential to validate these hypotheses and refine genotype-driven risk stratification in HHT.

### Age effects

Age-related patterns were evident in our analysis: older patients reported more frequently gastrointestinal bleeding and hepatic AVMs, whereas younger patients had fewer of these complications and sometimes lacked telangiectasis. Of note, our multivariate analysis showed that each year of age was associated with a 3.0% decrease in the odds of bAVMs (Table 5), while hepatic AVMs showed a 2.6% increase in the odds per year after adjustment for other covariates (Table 6). Bleeding from GI telangiectasis typically began in later adulthood and were rare before the age of 50. Likewise, hepatic AVMs tend to be identified around the fifth decade of life [24], reflecting cumulative visceral vascular changes over time. This pattern is consistent with the known age-dependent penetrance of HHT [24]. Prior studies have similarly found that hepatic vascular malformations are significantly more frequent in older HHT patients [25].

Increasing age, when stratified into three age groups, was not associated with a higher prevalence of neurological findings; however, this result should be interpreted with caution given the limited sample size of the study.

In contrast, epistaxis and mucocutaneous telangiectasis usually start earlier but continue to accumulate over time [4]. These findings demonstrate the progressive nature of HHT: younger individuals may be asymptomatic or have only nosebleeds, while older patients are more likely to manifest multi-organ involvement and complications in line with reports of age-dependent HHT progression [25].

### Gender differences

We also observed sex-related differences in clinical expression of HHT. Our analysis showed higher rates of focal neurological deficits (impairments of nerve, spinal cord, or brain function affecting specific rather than global body regions)in male patients, while strokes, hemorrhages, and epileptic seizures were similar across the groups. The literature on gender-related differences across patients with HHT is scarce. In 2020, a series by Mora-Luján et al. found that women tend to have more severe hepatic involvement than men. Roughly 60% of women showed liver AVMs on imaging (versus 47% of men) [26]. However, this difference was not significant in our cohort (*p* = 0.392). In the Mora-Luján et al. series, females also reported higher liver disease risk scores and more frequent need for liver transplantation [24, 26].

The same contradiction also emerges when evaluating pulmonary AVMs rates: Mora-Luján et al. noted women had a higher prevalence of pulmonary AVMs (35% vs. 23% in men) [26], whereas men showed more duodenal telangiectasia (21% vs. 10%) and tended to require emergency care visits more often [26]. These differences have been attributed in part to hormonal and in part to physiologic factors. Nevertheless, our study did not show these patterns (*p* = 1.00). Future research is needed to address this gap and guide sex-tailored surveillance protocols.

Interestingly, our finding of more neurologic complications (*p* = 0.009) in males diverges from the report by Mora-Luján et al., who found similar rates of ischemic stroke despite gender (4–5% in each). These discrepancies may be attributable to differences in sample size and familial clustering within the study populations.

Overall, aside from these exceptions, HHT is phenotypically equal in both sexes.

### Limitations

This study has several limitations. First, its retrospective design and the lack of long-term follow-up does not allow speculations on temporal evolution of cerebrovascular patterns. Additionally, restricting inclusion to patients with available brain MRI inevitably introduces selection bias, as asymptomatic individuals may not have undergone cerebral imaging and therefore would not have been captured in the study cohort. In addition, the study population was entirely Caucasian, which limits the generalizability of the findings to other populations.

We also acknowledge a potential limitation related to the use of the Curaçao score as a covariate in the logistic regression models. Because the presence of visceral vascular malformations contributes only one point to the Curaçao diagnostic criteria, its inclusion could introduce incorporation bias when modeling vascular outcomes. For this reason, the Curaçao score was excluded from the final multivariable analyses. Several univariate associations were based on very small subgroup sizes, in some cases involving a single mutation carrier with a lesion, raising concerns for potential false-positive results. Additionally, the relatively limited sample size reduced the stability of some multivariable models, precluding the construction of more complex or fully adjusted models. Nevertheless, the rarity of the condition inherently limits statistical power, warranting cautious interpretation of these results.Finally, future studies incorporating familial clustering within multivariable models are needed to further validate the association between specific genetic variants and brain AVMs identified in this study.

## Conclusion

In HHT, age and gene-level effects are robust predictors of systemic AVM involvement, whereas mutation-specific associations remain difficult to evaluate. Larger multicenter studies are needed to validate variant-level risk and support personalized surveillance strategies.

## Data Availability

Available upon request by e-mailing the corresponding author.
